# Impact of partial-volume correction in oncological PET studies: a systematic review and meta-analysis

**DOI:** 10.1007/s00259-017-3775-4

**Published:** 2017-08-04

**Authors:** Matthijs C. F. Cysouw, Gerbrand M. Kramer, Linda J. Schoonmade, Ronald Boellaard, Henrica C. W. de Vet, Otto S. Hoekstra

**Affiliations:** 1 Department of Radiology and Nuclear Medicine, VU University Medical Centre, P.O. Box 7057, 1007 MB Amsterdam, Netherlands; 2 Department of Medical Library, VU University Medical Centre, Amsterdam, Netherlands; 3 Department of Nuclear Medicine & Molecular Imaging, University Medical Centre Groningen, Groningen, Netherlands; 4 Department of Epidemiology and Biostatistics, VU University Medical Centre, Amsterdam, Netherlands

**Keywords:** Pet, Partial-volume effect, Partial-volume correction, Oncology, Quantification

## Abstract

**Purpose:**

Positron-emission tomography can be useful in oncology for diagnosis, (re)staging, determining prognosis, and response assessment. However, partial-volume effects hamper accurate quantification of lesions <2–3× the PET system’s spatial resolution, and the clinical impact of this is not evident. This systematic review provides an up-to-date overview of studies investigating the impact of partial-volume correction (PVC) in oncological PET studies.

**Methods:**

We searched in PubMed and Embase databases according to the PRISMA statement, including studies from inception till May 9, 2016. Two reviewers independently screened all abstracts and eligible full-text articles and performed quality assessment according to QUADAS-2 and QUIPS criteria. For a set of similar diagnostic studies, we statistically pooled the results using bivariate meta-regression.

**Results:**

Thirty-one studies were eligible for inclusion. Overall, study quality was good. For diagnosis and nodal staging, PVC yielded a strong trend of increased sensitivity at expense of specificity. Meta-analysis of six studies investigating diagnosis of pulmonary nodules (679 lesions) showed no significant change in diagnostic accuracy after PVC (*p* = 0.222). Prognostication was not improved for non-small cell lung cancer and esophageal cancer, whereas it did improve for head and neck cancer. Response assessment was not improved by PVC for (locally advanced) breast cancer or rectal cancer, and it worsened in metastatic colorectal cancer.

**Conclusions:**

The accumulated evidence to date does not support routine application of PVC in standard clinical PET practice. Consensus on the preferred PVC methodology in oncological PET should be reached. Partial-volume-corrected data should be used as adjuncts to, but not yet replacement for, uncorrected data.

**Electronic supplementary material:**

The online version of this article (doi:10.1007/s00259-017-3775-4) contains supplementary material, which is available to authorized users.

## Introduction

Positron-emission tomography (PET) enables in vivo assessment of metabolic and intracellular processes. Whereas in clinical practice, PET is predominantly used to qualitatively assess tracer uptake, PET(/computed tomography [CT]) may also serve as a surrogate quantitative biomarker of, for example, tumor metabolism and proliferation. The application of quantitative tumor assessment methods for distinguishing benign from malignant lesions, staging, prognostication, and determining or predicting response to therapy has garnered increasing interest [1–4].

Accurate quantification of metabolic volumes <2–3× the spatial resolution of PET is hampered by partial-volume effects, leading to underestimations of standardized uptake value (SUV), and possibly compromising lesion detection [5, 6]. Many methods for partial-volume correction (PVC) have been advocated [7]. The simplest technique uses recovery coefficients (RC) obtained from phantom experiments under the assumption that true metabolic volume is known and that lesions are spherically shaped with homogeneous uptake. More sophisticated methods have been developed, but all suffer from limitations [7, 8]. Voxel-wise resolution recovery methods, incorporating the point spread function (PSF) within iterative reconstruction [9] (PSF reconstruction) or performing post-reconstruction iterative deconvolution [10], could improve both qualitative and quantitative reads. To date, consensus on standardized application of PVC in oncological PET/CT studies is lacking, and perhaps as a consequence PVC is not yet routinely applied. In fact, most current clinical quantitative PET studies merely exclude small lesions (e.g. <2 cm in diameter), as recommended in the PET Response Criteria in Solid Tumors (PERCIST) criteria [3].

The clinical impact of PVC in an oncological setting, and thus the need for standardized application, is not yet fully elucidated [7]. We performed a systematic review and meta-analysis to assess the impact of PVC in clinical PET studies, focusing on diagnosis, staging, prognostication, and response assessment.

## Materials and methods

### Search strategy

This systematic review was conducted in accordance with the Preferred Reporting Items for Systematic Reviews and Meta-Analysis (PRISMA) statement. A comprehensive search (Supplemental Tables 1 and 2), in collaboration with a medical librarian (LJS), was performed in PubMed and Embase.com from inception to May 9, 2016. Both controlled terms (MesH in PubMed, Emtree in Embase) and free-text terms were included in the search. The following were used (including synonyms and closely related words) as index terms or free-text words: ‘positron-emission tomography or ‘PET’ and ‘partial volume correction’ or ‘point spread function reconstruction’ and ‘neoplasms’ or ‘cancer’.

### Selection process

Abstracts and titles of all studies retrieved from the search were independently screened by two researchers (MCFC and GMK). Afterwards, eligible articles were studied in full text. In case of differences in judgment, consensus was reached through discussion. Cross-referencing was performed to further identify relevant articles.

The following were included: studies applying PVC in clinical PET studies, using oncological patients, reporting PET data with and without PVC, and investigating clinical impact of PVC on either diagnosis, staging, prognostication (reporting survival data), or response assessment.

Exclusion criteria were as follows: reviews, letters, editorials, conference abstracts, case reports, full text not available or not in English, no adequate reference data, no description of or reference to PVC method, combined PVC and motion blur correction method, or patient cohort overlapping with another included study.

### Quality assessment

The quality of included articles was assessed (independently by MCFC and GMK) according to the QUADAS-2 [11] (*n* = 25) or QUIPS [12] (*n* = 12) tools. QUADAS-2 assesses bias and applicability of diagnostic studies, whereas QUIPS assesses bias of studies investigating prognostic factors. Staging and response assessment studies were assigned to either of the quality assessment tools. Consensus was reached through discussion.

### Data extraction and meta-analysis

Both researchers independently extracted results regarding impact of PVC on diagnostic accuracy (for diagnosis and staging), prediction of survival (for prognostication), and response assessment. Measures of diagnostic accuracy were derived with and without PVC. If test characteristics were described for subgroups, overall measures of accuracy were calculated when possible. When *p*-values of differences in accuracy between uncorrected and PVC data were not reported, these differences were deemed not statistically significant. Descriptive data regarding cancer type, number of patients, lesion sizes, scanner type, and PVC method were also extracted. Unless stated otherwise, we presented data on SUV quantification.

Diagnostic studies on the same topic were pooled using bivariate random effects meta-regression analysis, which is the recommended method for meta-analysis of diagnostic studies [13]. This method provides summary estimates of sensitivity and specificity with 95% confidence intervals, taking into account the correlation between sensitivity and specificity and heterogeneity in results between studies. We tested for differences in overall diagnostic accuracy between different diagnostic tests using a likelihood ratio test, comparing models that included and excluded a covariate for the diagnostic test. For illustrative purposes, summary receiver operating characteristic (ROC) curves were calculated according to the Moses-Littenberg method [14]. We used Stata software (version 14; StataCorp LP, College Station, TX) for statistical analyses.

## Results

### Study selection

**Fig. 1:**
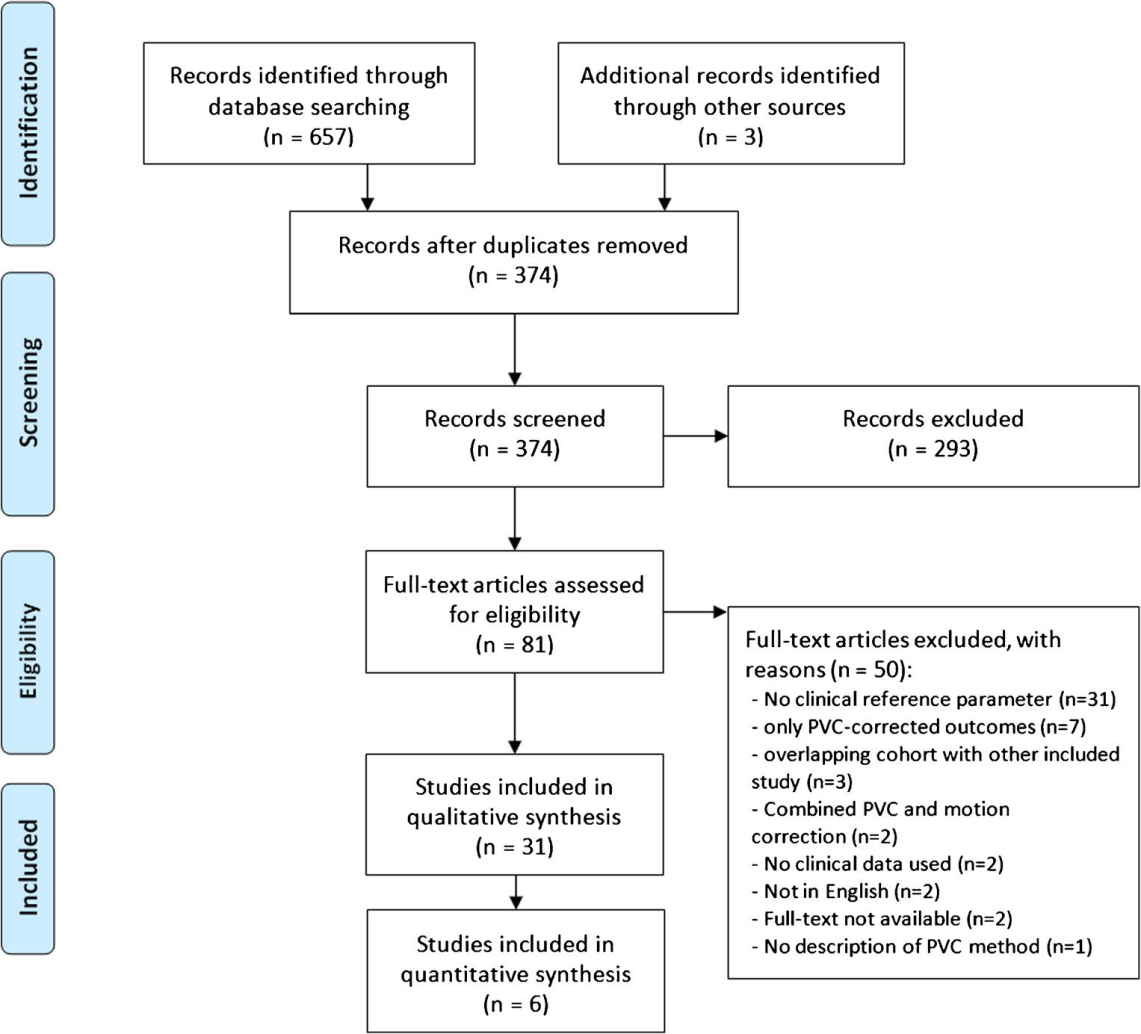
PRISMA flowchart

Pubmed and EMBASE searches yielded 371 potentially eligible studies (Fig. 1). Three additional studies were found through reference screening. Two hundred and ninety-three abstracts were excluded based on eligibility criteria, leaving 81 for full-text screening. For 19 (5.1%) abstracts, judgments were conflicting, and consensus was reached through discussion. After full-text review, 31 studies met eligibility criteria (Fig. 1). Studies on diagnosis (*n* = 10), staging (*n* = 10), prognostication (*n* = 6), and response assessment (*n* = 5) are presented in Tables 1, 2, 3, and 4, respectively. Supplemental Table 3 contains the PVC and tumor delineation methodologies, reconstruction settings, full-width-at-half-maximum values, and voxel sizes of each included study. Thirty studies used ^18^F-FDG as radiopharmaceutical, one study used ^18^F-choline.

**Table 1:** Eligible diagnostic studies, in chronological order

Ref.	No. of patients	Target lesions	No. and type of lesions	Lesion sizes (mm)^a^	Cut-off	Non-PVC PVC	Reference test(s)	Effect on test performance?
[15]	73	Breast tumors	51 M, 46 B	25 ± 9 (B), 27 ± 17 (M)	Data-driven	2.1 2.1	Histology	Sens ↑ 69 to 81% Spec = 90%
[16]	27	Malignant lymphoma	NS	Median 18 (range 8–53)	NA		Follow-up / biopsy	NA
[17]	127	Pulmonary nodules	86 M, 41 B	33 ± 23	Pre-defined	2.5 2.5	Histology	Sens = 94% Spec ↓ 76 to 67%
[18]	47	Pulmonary nodules	36 M, 11 B	21.6 ± 9.7	Pre-defined	2.5 2.5	Follow-up / biopsy	Sens ↑ 72 to 97% Spec ↓ 82 to 73%
[19]	60	Pulmonary nodules	46 M, 14 B	26.3 ± 15.8 (M), 20.4 ± 10.4 (B)	Pre-defined	2.5 2.5	Histology	Sens ↑ 87 to 98% Spec ↓ 21 to 14%
[20]	265	Pulmonary nodules	72 M, 193 B	<10 (*n* = 32), 10–15 (*n* = 57), 16–30 (*n* = 176)	Pre-defined	2.5 2.5	Follow-up / biopsy	Sens ↑ 65 to 90% Spec ↓ 92 to 80%
[21]	46	Pulmonary nodules	26 M, 23 B	20 ± 7 (M), 13 ± 5 (B)	Data-driven	2.4 2.9	Follow-up / biopsy	Sens ↑62 to 73% Spec = 80%
[22]	42	NHL	26 aggressive 16 indolent	32.4 ± 18.3 (aggressive), 21.9 ± 10.3 (indolent)	Data-driven	9.5 11.2	Histology	Sens = 81% Spec ↓ 81 to 63%
[23]	131	Pulmonary nodules	86 M, 45 B	29.1 ± 18.1	Pre-defined	2.5 2.5	Histology	Sens ↓ 89 to 88% Spec ↓ 51 to 42%
[24]	22	Lymph nodes	8 KFD, 14 NHL	13.8 ± 5.4 (KFD), 25.4 ± 11.8 (indolent), 29.7 ± 18.8 (aggressive)	NA		Histology	NA

*M* malignant, *B* benign, *NHL* non-Hodgkin lymphoma, *KFD* Kikuchi-Fujimoto disease, *NA* not applicable, *NS* not specified, *Sens* sensitivity, *Spec* specificity

^a^Sizes are presented as mean ± SD, unless stated otherwise

**Table 2:** Eligible studies evaluating staging, in chronological order

Ref.	No. of patients	Cancer type	No. and type of lesions	Lesion sizes (mm)^a^	T/N/M	Cut-off	Non-PVC PVC	Method of staging	Effect on test performance?
[25]	178	NSCLC	NS	Range 18 ± 5 to 44 ± 20	TNM	NA		Imaging / surgery / pathology	NA
[26]	7	Thyroid (mLN)	15 M, 24 B	NS	N	Data-driven	4.0 10.0	Imaging / pathology	Sens = 100% Spec ↑ 92 to 100%
[27]	52	Breast	NS	NS	N	NS	NS	Imaging	Sens ↑ 75 to 86% Spec ↓ 87 to 83%
[28]	58	NSCLC	201	7.2 ± 1.7 (<10 mm), 19.2 ± 1.05 (≥10 mm)	N	NA		Pathology / imaging / clinical	Sens ↑ 78 to 97% Spec ↓ 71 to 58%
[29]	35	Lung	NS	30 (range 8–79)	TNM	NA		Imaging / pathology	NA
[30]	50	Breast (mLN)	NS	8.2 ± 4.3	N	NA		Pathology	Sens ↑ 76 to 85% Spec ↓ 75 to 69%
[31]	32	HNSCC (mLN)	18 M, 39 B	1.14 ± 1.38 mL (M), 0.64 ± 0.93 mL (B)	N	Data-driven	NS	Pathology	Sens ↑ 57 to 64% Spec ↑ 71 to 76%
[32]	71	Nasopharyngeal (mLN)	35 M, 53 B	<6 (*n* = 55) 6–6.9 (*n* = 7) ≥7 (*n* = 26	N	Pre-defined	2.5 n.s	Imaging	Sens ↑ 77 to 94% Spec ↓ 89 to 59%
[33]	39	Prostate	49 prostatic, 43 nodal	NS	TN	Data-driven	2.4 5.0	Pathology / imaging / PSA	Sens ↓ 90 to 84% Spec = 73%
[34]	38	Colorectal	32 M, 115 B	NS	N	NA		Surgery / pathology	Sens ↑ 53 to 66% Spec = 99.1%

*M* malignant, *B* benign, *NSCLC* non-small cell lung cancer, *HNSCC* head and neck squamous cell carcinoma, *mLN* lymph node metastases, *NA* not applicable, *NS* not specified, *PSA* prostate-specific antigen, *Sens* sensitivity, *Spec* specificity

^a^Sizes are presented as mean ± SD, unless stated otherwise

**Table 3:** Eligible studies evaluating prognostication, in chronological order

Ref.	No. of patients	Cancer type	No. of lesions	Spectrum of tumor sizes (mm)^a^	Effect on prognostication?
[35]	145	NSCLC	NS	Median 30 (range 10–110)	Not improved
[36]	52	Esophageal	NS	NS	Not improved
[37]	50	Esophageal	NS	39.9 ± 36.1 mL	Not improved
[38]	191	NSCLC	NS	Median 23 (range 10–36)	Not improved
[39]	19	HNC	19	15.2 ± 5.0	Improved
[40]	19	HNC	19	15 ± 5	Improved for subgroup

*NSCLC* non-small cell lung cancer, *mLN* lymph node metastases, *HNC* head and neck cancer, *NS* not specified

^a^Sizes are presented as mean ± SD, unless stated otherwise

**Table 4:** Eligible studies evaluating response assessment, in chronological order

Ref.	No. of patients	Cancer type	No. of lesions	Spectrum of tumor sizes (mL)^a^	Reference test	Effect on response assessment?
[41]	35	LABC	NS	NS	Clinical + pathologic	Not improved
[42]	51	Breast	NS	Median 14 (range 2–227)	Pathologic	Not improved
[43]	28	LARC	NS	Median 23 (range 2–397)	Pathologic	Not improved
[44]	40	mCRC	101	34.4 ± 66.4	RECIST	Worsened
[45]	19	NSCLC	24	Median 6.95 (range 2.2–46)	Clinical	PERCIST classification improved in 5 lesions, confirmed in follow-up

*LABC* locally advanced breast cancer, *LARC* locally advanced rectal cancer, *NSCLC* non-small cell lung cancer, *mCRC* metastatic colorectal cancer, *RECIST* Response Evaluation Criteriain Solid Tumors, *PERCIST* PET Response Criteria in Solid Tumors, *NS* not specified

^a^Sizes are presented as mean ± SD, unless stated otherwise, at baseline

### Quality assessment

For extensive descriptions of the QUADAS-2 and QUIPS scoring criteria, we refer to their respective primary publications [11, 12].

**Fig. 2:**
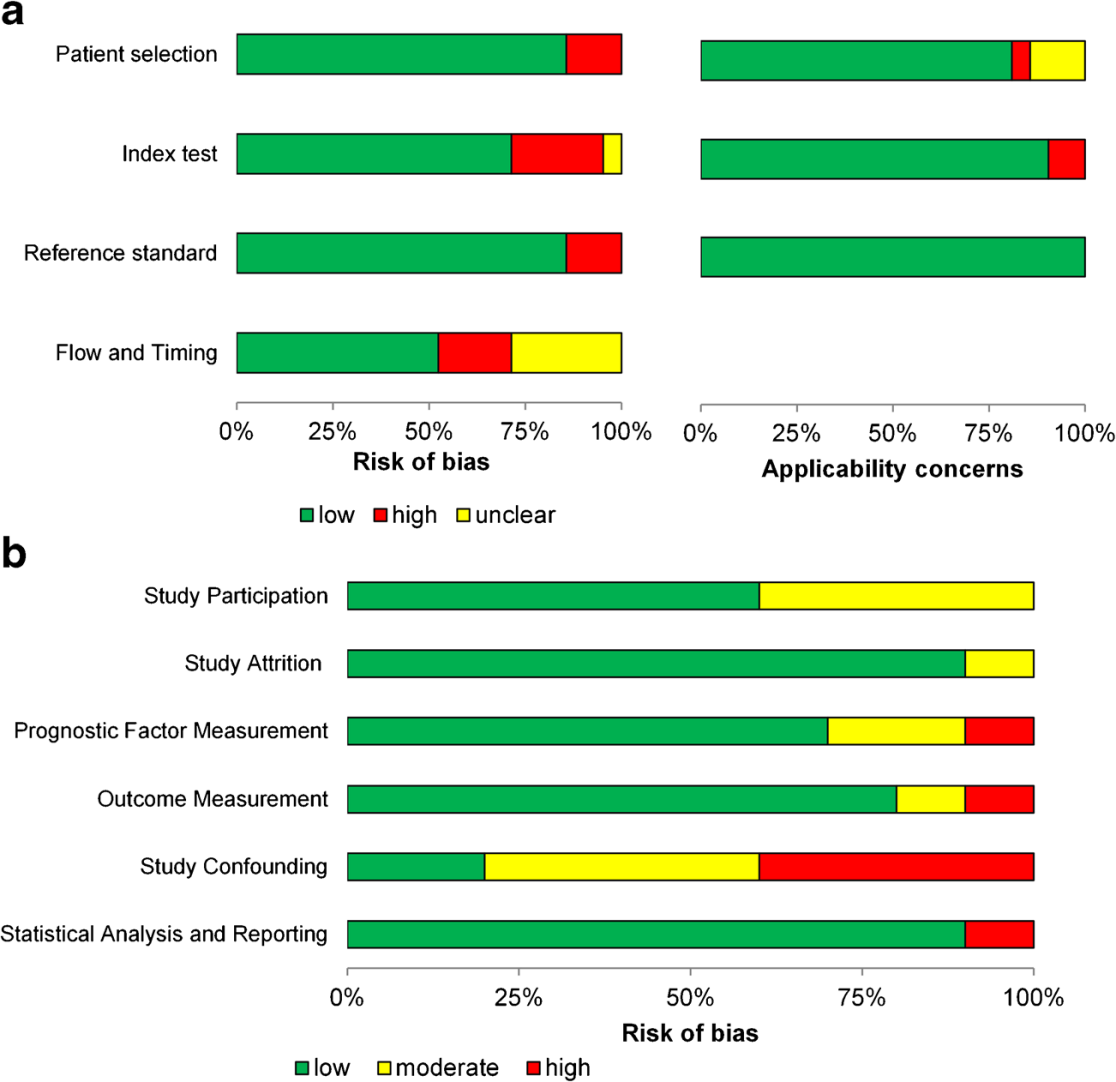
Results of quality assessment according to QUADAS-2 (**a**) and QUIPS (**b**) tools

Considering QUADAS-2 (Fig. 2a), the ‘reference standard’ and ‘patient selection’ items resulted in low risk of bias (high risk of bias in 14% of studies for either item). Elevated risk of bias for the ‘reference standard’ item was caused by use of multiple reference tests within the same study. Risk of bias in the index test was high in 24% of studies due to the use of data-driven instead of pre-defined SUV cut-offs. Applicability concerns regarding patient selection were mainly caused by large tumor size spectra and unspecified tumor sizes.

Using QUIPS (Fig. 2b), low risk-of-bias scores were found in the majority of the studies for the items measurement of outcome and prognostics factors, study attrition, and statistical analysis and reporting. Several studies did not adequately investigate potential factors of study confounding, which resulted in a moderate risk of bias in 40% of studies and high risk of bias in 40% of studies. Unclear descriptions of included patient cohorts (‘study participation’ item) resulted in moderate risk of bias in 40% of included studies.

### Diagnosis

Impact of PVC on diagnosis (Table 1, *n* = 10) was investigated for pulmonary nodules (*n* = 6), breast lesions (*n* = 1), and lymphoma (*n* = 3). PVC included the RC method (*n* = 9) and CT volume-based PVC (*n* = 1). All studies reported lesion sizes. One study stratified both uncorrected and PVC data for lesion size in secondary analysis.

**Fig. 3:**
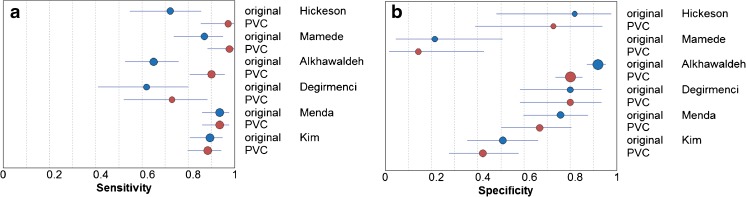
Forest plots presenting sensitivity (**a**) and specificity (**b**) with 95% CI of discrimination between benign and malignant pulmonary nodules with ^18^F-FDG-PET

**Fig. 4:**
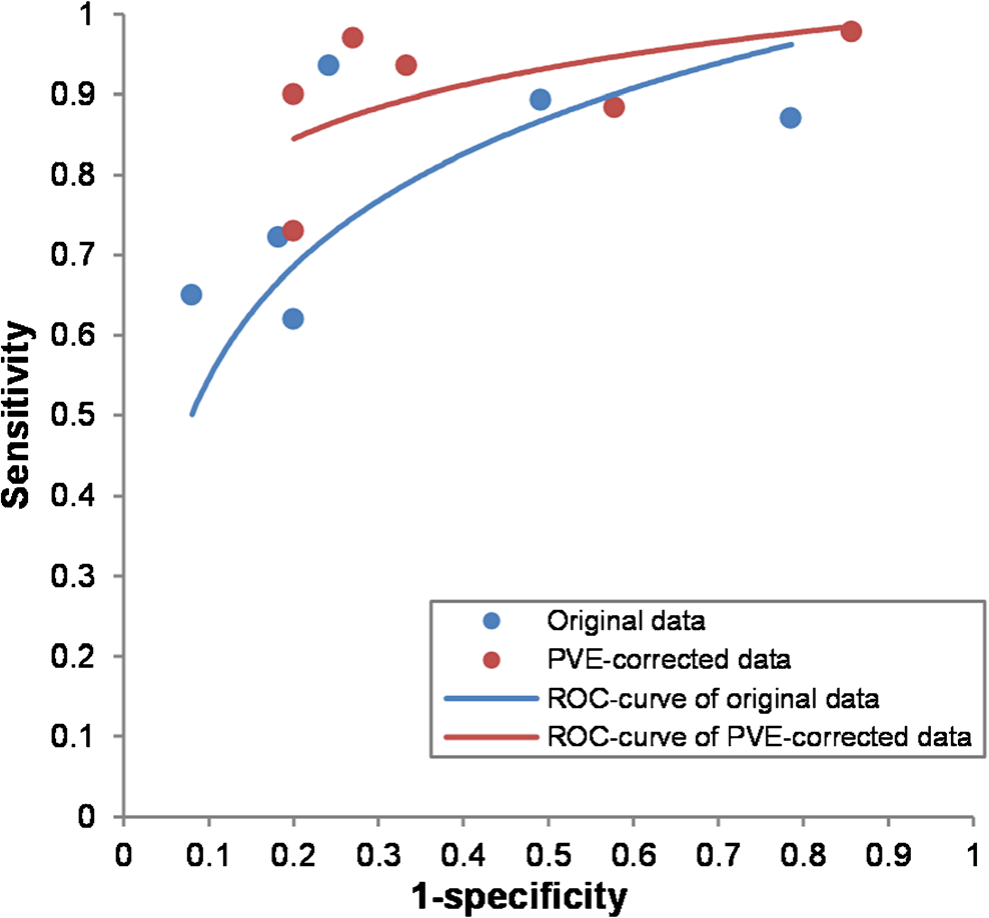
Summary ROC curves of discrimination between benign and malignant pulmonary nodules with ^18^F-FDG-PET

**Fig. 5:**
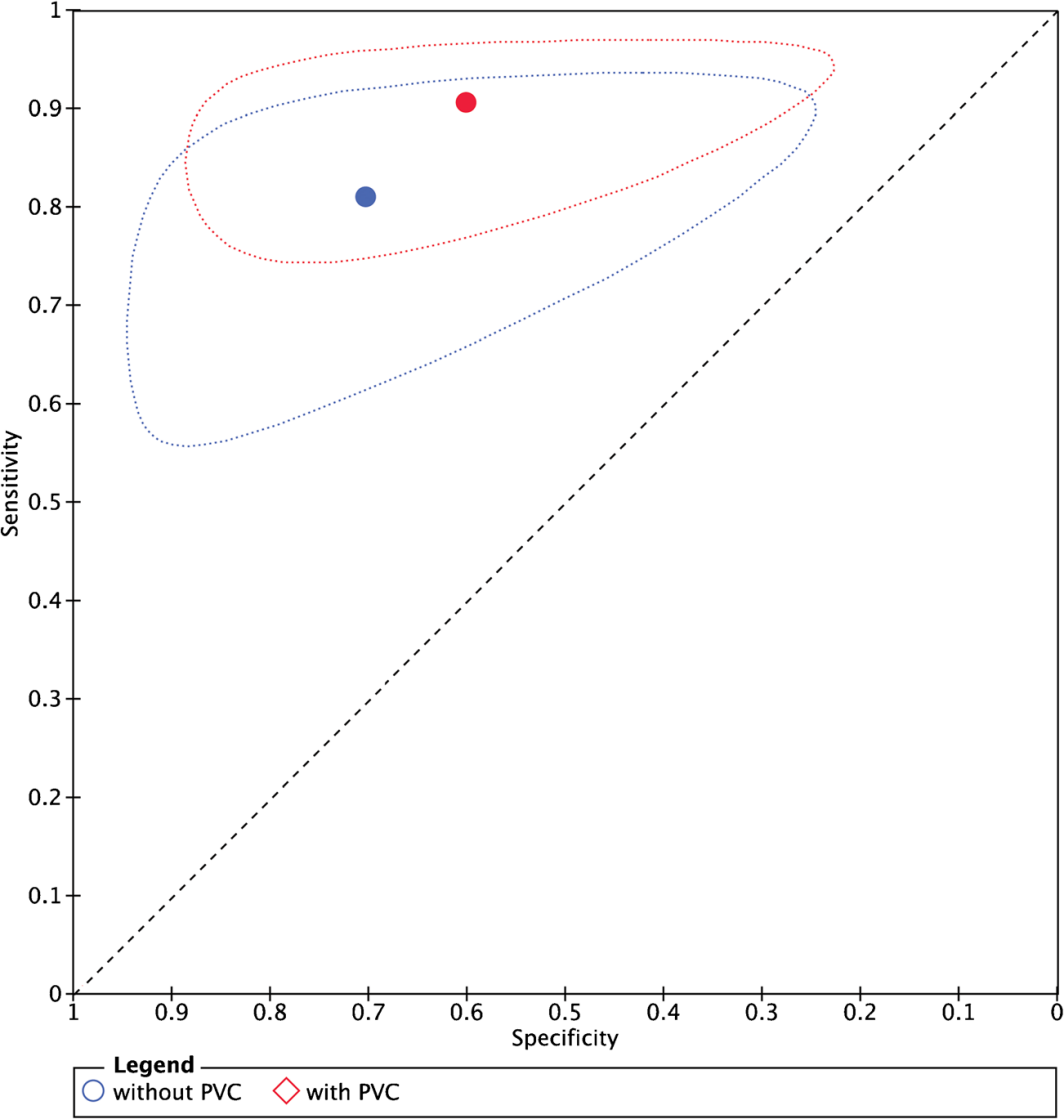
Summary sensitivity and specificity with 95% confidence region of discrimination between benign and malignant pulmonary nodules with ^18^F-FDG-PET

The six studies evaluating diagnostic accuracy of PET for pulmonary nodules were pooled (Table 1, Figs. 3 and 4), and included a total of 352 malignant and 327 benign lesions [17–21, 23]. Prevalence of malignancy ranged from 27 to 77% (mean 57%). Five studies applied an RC method for PVC, one study applied a CT volume-based correction. Thresholds of PET positivity were predefined in 5/6 studies and data-driven in 1/6 studies. Predefined thresholds were similar for uncorrected and PVC data. Three studies used SUV 2.5 as predefined threshold [19, 20, 23]. One study used SUV 2.0 and 2.5 as thresholds [17]. One study used SUV 1.5, 2.0, 2.5, and 3.0 as thresholds [18]. In case of multiple predefined thresholds, results of the SUVmax 2.5 threshold were used in meta-analysis (SUVmean for PVC data in Hickeson et al.) since this was reported in all 5 studies with predefined SUV thresholds. One study used data-driven thresholds specifically for uncorrected (SUV 2.4) and PVC data (SUV 2.9) [21]. Pooled sensitivity and specificity of uncorrected data were 81% (95% CI 70–89) and 70% (95% CI 48–86), respectively (Fig. 5). Pooled sensitivity and specificity of partial-volume-corrected data were 91% (95% CI 83–95) and 60% (95% CI 37–79), respectively (Fig. 4). No significant change in diagnostic accuracy after PVC was found (*p* = 0.222), using the SUV thresholds as described above. One of the pulmonary studies (by Hickeson et al.) stratified both uncorrected and corrected data for lesion size [18]. The authors observed that for lesions <2 cm, accuracy increased from 59 to 85% using an SUV cut-off 2.5, while for lesions >2 cm, accuracy changed from 95 to 100%.

With diagnosis of breast lesions, using data-driven SUVmean thresholds of 2.1 for PVC and non-PVC, at a fixed specificity of 90%, PVC increased sensitivity from 69 to 81%, but the impact on accuracy was not statistically significant [15]. In discriminating between aggressive and indolent non-Hodgkin lymphoma (NHL), PVC decreased specificity without affecting sensitivity [22]. Similarly, PVC did not improve differentiation between high- and low-grade NHL [16]. PVC also enabled differentiation between indolent NHL and Kikuchi-Fujimoto disease [24].

### Staging

Studies evaluating the effect of PVC on staging (Table 2, *n* = 10) included lung (*n* = 3), breast (*n* = 2), thyroid (*n* = 1), head and neck squamous cell (*n* = 1), nasopharyngeal (*n* = 1), prostate (*n* = 1), and colorectal cancer (*n* = 1). Applied PVC methods included the RC method (*n* = 4), PSF reconstruction (*n* = 4), iterative deconvolution (*n* = 1) and geometric transfer matrix (*n* = 1). Most of these studies did not specify SUV thresholds of test positivity for uncorrected and PVC data. Four studies did not specify lesions sizes. One study stratified both uncorrected and PVC data for lesion size in secondary analysis.

In non-small cell lung cancer (NSCLC) patients the association between primary tumor SUVmax and overall TNM stage disappeared after PVC [25]. For nodal staging using SUV, non-significant trends of increased accuracy for breast, head and neck squamous cell, and thyroid cancer (from 80%, 66% and 95% to 84%, 71% and 100%, respectively) [26, 27, 31], and decreased accuracy for nasopharyngeal and prostate cancer (from 84% and 85% to 73% and 80%, respectively) were observed [32, 33]. The study investigating accuracy of nodal staging of nasopharyngeal cancer did observe a large increase in accuracy, from 14 to 71%, when stratifying for lesion size (6–7 mm diameter) [32].

With visual image interpretation, PSF reconstruction tended to increase accuracy of nodal staging in NSCLC, breast, and colorectal cancer (not statistically significant) compared to non-PSF reconstruction (from 76%, 76%, and 89% to 84%, 80%, and 92%, respectively) [28, 30, 34]. Another study found no significant difference in lung cancer (several types) overall staging accuracy between non-PSF and PSF reconstruction [29].

### Prognosis

Impact of PVC on prognostication (Table 3, *n* = 6) was investigated for NSCLC (*n* = 2), esophageal (*n* = 2), and head and neck cancer (*n* = 2). Applied PVC methods were the RC method (*n* = 4), iterative deconvolution (*n* = 1), and mask-based PVC (*n* = 1). Only prognostic studies providing survival data were included. One study did not specify lesion sizes. None of the studies stratified results on PVC for lesion size in secondary analysis.

PVC did not alter the association of SUVmax with disease-free survival of NSCLC (various histological types) patients in multivariate analysis [35, 38]. Similarly, in NSCLC patients (various histologic types), PVC did not alter the ROC area under the curve of primary tumor SUVmax to differentiate between groups of patients in terms of disease-free and overall survival [38]. Primary tumor SUVs, regardless of PVC, were insufficient as prognostic markers in esophageal (adeno- and squamous cell) cancer in univariate and ROC analysis [36, 37]. In head and neck cancer patients, partial-volume-corrected SUV was significantly different between patient groups stratified according to disease-free survival, whereas uncorrected SUV was not [39]. In univariate analysis, PVC did not affect predictive value of head and neck cancer primary tumor SUV on local recurrence-free survival, distant metastasis-free survival, and disease-free survival, but did allow for prediction of distant metastasis-free survival in a subgroup of patients with PET-positive lymph nodes [40].

### Response assessment

Impact of PVC on response assessment (Table 4, *n* = 5) was investigated for breast (*n* = 2), rectal (*n* = 1), colorectal (*n* = 1), and NSCLC (*n* = 1). Applied PVC methods included the RC method (*n* = 2), iterative deconvolution (*n* = 2), and both RC method and iterative deconvolution (*n* = 1). One study did not specify lesion sizes. None of the studies stratified results on PVC for lesion size in secondary analysis.

For locally advanced breast cancer [41], regardless of PVC primary tumor FDG, metabolic rate was not able to differentiate between clinical and pathologic responders and non-responders during neoadjuvant chemotherapy (after 2 months). In another study in breast cancer patients PVC did not significantly change prediction of pathologic response with primary tumor SUV during neoadjuvant therapy (after two cycles) [42]. In locally advanced rectal cancer patients treated with (preoperative) chemoradiotherapy, PVC had no impact on histopathological response prediction, at baseline or after 1 or 2 weeks of therapy [43]. In patients with metastatic colorectal cancer PVC significantly reduced the ROC area under the curve of SUV in discriminating between responders and non-responders after 2 weeks of chemotherapy, as defined with RECIST [44]. In NSCLC patients treated with radio- or radiochemotherapy, PVC changed PERCIST [3] classification of response in 5/24 lesions, which were verified as correct alterations in clinical follow-up [45].

## Discussion

Quantification of functional tumor characteristics with PET is considered to be useful in clinical oncology, and often uses semi-quantitative analyses, resulting in SUVs. Unfortunately, partial-volume effects are known to cause underestimation of tumor activity, and hence the necessity of PVC for accurate semi-quantitative reads for small lesions is well recognized [5]. However, many factors affect its accuracy and potentially hamper its optimal usage. Perhaps as a consequence, its resulting advantage in oncological PET studies is not yet evident. Additionally, the lack of consensus on the preferred PVC and delineation method may result in suboptimal results and could hamper comparisons between studies. This review discusses the clinical impact of PVC and provides recommendations for specific research questions and analyses to be included in future studies applying PVC.

When applied to diagnosis of primary lesions and (mainly nodal) staging, PVC often yielded higher sensitivity at the expense of specificity (Tables 1 and 2 and Figs. 3 and 4), which is an obvious consequence when using the same test positivity SUV thresholds for uncorrected and PVC data. In the subset of studies which allowed statistical pooling (679 lesions), meta-analysis showed that PVC did not significantly alter the overall diagnostic accuracy in characterizing pulmonary lesions with PET (Fig. 5). When estimating the effect of PVC, the optimal trade-off between sensitivity and specificity (the SUV threshold of test positivity) may be different for PVC and uncorrected data. At an exploratory level, one should define this cut-off for either method. Of note, Degirmenci et al. (on pulmonary nodules) used data-driven SUV cut-offs of 2.4 and 2.9 for uncorrected and PVC data, respectively, which yielded a specificity fixed at 80%, with sensitivity of 62 and 73% for uncorrected and PVC data, respectively [21]. We performed a similar analysis using the (individual patient) data from Hickeson et al. [18]. At a predefined SUV cut-off of 2.5, PVC decreased specificity and increased sensitivity (Table 1). However, when applying cut-offs of 2.55 and 2.8 (as derived from ROC analysis) for uncorrected and PVC data, respectively, PVC increased sensitivity from 72 to 94%, while specificity remained constant at 91%. This further demonstrates that PVC may indeed increase diagnostic accuracy when SUV cut-offs are adequately adapted for this correction. Obviously, each proposed threshold requires external validation.

Another explanation for the limited impact of PVC on diagnostic accuracy as published in the literature may relate to the size spectra of included lesions, with the distribution of benign and malignant lesions therein. When performing PVC analysis simultaneously on all lesions, both large and small, the overall impact of PVC on diagnostic accuracy will be diminished. Indeed, several studies demonstrated a high impact of PVC on accuracy for small lesions (when stratifying for lesion size), but less so when including all lesions regardless of size [18, 32]. Therefore, we suggest that investigators stratify diagnostic performance results for lesion size in secondary analyses. However, since partial-volume effects are not merely size-dependent, but are also affected by lesion contrast and shape, reliable classification of lesions that are (most) affected by partial-volume effects will be difficult. In our previous simulation study, we observed that for high-contrast spherical lesions, partial-volume effects started to occur below 3-cm diameter [8]. A practical approach for stratification would thus be to stratify results using a 3-cm lesion diameter or a 14-mL metabolic volume cut-off (corresponding to a 3-cm-diameter sphere). Even though larger lesions may also be somewhat affected by partial-volume effects, depending on their shape and contrast, such a size cut-off will ensure that lesions that are most affected by partial-volume effects are separated. Another approach would be to plot the percentage increases in SUV after PVC as a function of metabolic tumor volume to determine an appropriate size cut-off for stratification of results within studies (not possible when applying the RC method).

Regarding visual nodal staging, PSF reconstruction did not significantly alter accuracy, but tended to increase sensitivity in lung, breast, and colorectal cancer (Table 2) [28, 30, 34]. This may be attributed to improved qualitative reads, improved (small) lesion detection, and higher diagnostic confidence [28, 30, 34]. Therefore, it may be worthwhile to validate these higher-resolution reconstruction algorithms for use in clinical practice, especially for detection of small lymph node metastases and lesions embedded in high background activity such as in the liver or mediastinum. However, PSF reconstructions may suffer from Gibbs artifacts (overshoot in activity); moreover, they are known not to guarantee full signal recovery [9]. Also, further research into their impact on compliance with European Association of Nuclear Medicine (EANM) standards is needed to ensure equal scanner calibration in multicenter quantitative PET/CT studies, which may require an SUV harmonization procedure [46].

We found that PVC might improve prognostication in head and neck cancer [39, 40], but these studies did not stratify for the human papillomavirus status, a prognostic marker associated with lower tumor SUV and smaller metabolically active tumor volume (MATV) [47]. For future studies, please note that appropriate PVC may not necessarily improve prognostication with SUV, but instead may enable it to reflect its true prognostic value. For example, Vesselle et al. found that PVC mitigated the correlation between primary tumor SUV and overall survival in NSCLC patients, and they also observed that the correlation between SUV and overall TNM stage, which in essence is based on patient prognosis, disappeared after PVC, suggesting that the ‘prognostic value’ of uncorrected SUV was based on tumor volume rather than metabolic activity [5, 25, 48].

For response assessment, no conclusions regarding the effect of PVC can be made at this point due to the small number of heterogeneous studies. One included study demonstrated that after PVC PERCIST classification of response was altered for 5/24 NSCLC lesions during radio- or radiochemotherapy [45]. This is an important observation, since, conceptually, PVC may correct changes in SUV during treatment for changes in tumor volume and contrast, allowing for more appropriate PET-based classification of tumor response. Interestingly, two studies (excluded since no clinical verification was performed) demonstrated PVC to alter response classifications according to European Organisation for Research and Treatment of Cancer (EORTC) or PERCIST criteria in patients with bone metastases and NSCLC [39, 49]. In conclusion, future PET response assessment studies should include PVC to allow for metabolic response assessment, irrespective of tumor shrinkage or growth, and quantify its clinical impact.

To improve comparison of PVC’s impact between studies, consensus on the preferred combination of PVC and lesion delineation methodologies should be reached. Many PVC methods have been advocated, some specific for oncological application [5, 7, 50, 51]. Still, most studies in this review applied an RC method, a quite simple method assuming spherically shaped lesions, homogeneous activity distributions, and known tumor sizes. Using this method, even small errors in tumor size measurements may result in over- or underestimations of true SUVs. Also, size measurements are often CT-based, whereas partial-volume effects affect metabolic volumes, which may be different from anatomical tumor volume [52] (e.g. due to necrosis and treatment effects). In a previous phantom and simulation study we found that voxel-wise PVC methods such as iterative deconvolution may be preferred, since this only assumes approximate knowledge of PET/CT systems’ resolution kernel size, has low dependency on accurate delineation, and has only limited effect on precision [8]. Additionally, such a voxel-wise PVC method could allow for more accurate delineation of tumors [53] and, theoretically, heterogeneous tumor background. However, iterative deconvolution is known to increase image noise levels, which may require some form of a denoising algorithm to be applied [37]. Iterative deconvolution may be relatively easy to implement, and has been demonstrated to perform well using commonly applied background-adapted threshold-based delineation methods [8]. To date, iterative deconvolution has been applied predominantly by the same research group (Supplemental Table 3); more extensive clinical evaluation is warranted. Our previous phantom and simulation study showed that for lesions ≤10 mm in diameter, even with PVC, the acquisition of fully accurate results was not yet possible [8], which may contribute to the relatively low impact of PVC. Owing to heterogeneity between studies, the impact of chosen PVC methods on outcomes cannot be established in this review.

A limitation of this systematic review and the meta-analysis was the small number of studies included (only six diagnostic studies could be pooled; which is the maximum number of studies in any of the other subsections), with several sources of heterogeneity, such as the included lesion types, malignancy prevalence, lesion size spectra, PET acquisition and reconstruction settings, quantitation methods, and methodological quality. The overall study quality as assessed by QUADAS and QUIPS was good (Fig. 2), but more specific research questions regarding PVC are needed, along with more rigorous designs. Although it was a limitation in this review, the small number of retrieved studies applying PVC in oncology is also an important finding, highlighting the reduced application of PVC in recent decades.

## Recommendations

When applying PVC in studies investigating diagnostic accuracy, SUV thresholds should be redefined for corrected data. Also, results on test characteristics should be stratified for lesion size (using a 3-cm-diameter or 14-mL cut-off). In prognostication studies, partial-volume-corrected SUV may complement rather than substitute uncorrected SUV, and could be included separately in prognostic models. The impact of PVC on PERCIST classifications of response merits further investigation in prospective studies. For now, we recommend that lesions ≤10 mm in diameter should not be included in quantitative analyses until novel PVC methods proven to be efficacious for these lesions are available. To demonstrate dependency of results on the applied PVC methodology, studies comparing multiple methods in the same sample of patients are highly recommended. Both functional and volumetric semi-quantitative PET metrics should be investigated simultaneously, including SUVs, MATV, and their product TLG (see for example refs. [31, 37, 40, 42, 43]). Also, when PET is used for therapeutic dosimetry applications, e.g. for nuclide radiotherapy, PVC will likely improve estimates of tracer or radionuclide uptake, and thereby improve estimates of tumor radiation dose.

## Conclusion

The accumulated evidence to date does not support routine application of PVC in standard clinical PET studies. In meta-analysis of quantitative diagnostic PET studies, PVC did not increase diagnostic accuracy. Limitations of published studies include the lack of analysis stratified for size, limited exploration of the impact of alternative (SUV) thresholds of test positivity on diagnostic accuracy measures, and heterogeneity in applied PVC methodologies. For accurate and reproducible results on tumor uptake quantification, consensus on the preferred tumor delineation and PVC methodologies needs to be reached. Partial-volume-corrected metrics should be used as adjuncts to, but not yet replacement for, uncorrected data.

## Electronic supplementary material

Supplemental Table 1 (DOCX 11 kb)

Supplemental Table 2 (DOCX 11 kb)

Supplemental Table 3 (DOCX 72 kb)
