# Supplementary material for: Impact of partial-volume correction in oncological PET studies: a systematic review and meta-analysis
Source: Eur J Nucl Med Mol Imaging. 2017 Aug 4;44(12):2105–16. doi: 10.1007/s00259-017-3775-4 (PMC5656693; doi:10.1007/s00259-017-3775-4)
Supplement: Supplementary file 1 — (DOCX 11 kb) [file 259_2017_3775_MOESM1_ESM.docx]

**Supplemental Table 1) Search strategy in PubMed May 9, 2016 (read from bottom-up).**

| **Set** | **Search terms** | **Result** |
| --- | --- | --- |
| #4 | #1 AND #2 AND #3 | 322 |
| #3 | partial-volume effect*[tiab] OR partial volume effect*[tiab] OR partial-volume correction*[tiab] OR partial volume correction*[tiab] OR deconvolution*[tiab] OR recovery coefficient*[tiab] OR point spread function reconstruction*[tiab] OR PSF reconstruction*[tiab] OR PSF-reconstruction*[tiab] OR point-spread-function reconstruction*[tiab] OR resolution model*[tiab] OR resolution recover*[tiab] OR resolution model*[tiab] OR high definition*[tiab] OR high definition*[tiab] OR PSF model*[tiab] OR point-spread-function model*[tiab] OR point spread function model*[tiab] OR HD reconstruction*[tiab] | 10752 |
| #2 | "Neoplasms"[Mesh] OR oncolog*[tiab] OR cancer*[tiab] OR neoplasm*[tiab] OR tumour*[tiab] OR tumor*[tiab] OR carcinoma*[tiab] OR malignan*[tiab] OR metasta*[tiab] OR lesion*[tiab] OR lymphoma*[tiab] | 3931706 |
| #1 | "Positron-Emission Tomography"[Mesh] OR positron emission tomograph*[tiab] OR PET[tiab] OR PET/CT[tiab] OR PET-CT[tiab] OR FDG-PET*[tiab] OR 18F-FDG-PET*[tiab] | 90587 |
