# Supplementary material for: Impact of partial-volume correction in oncological PET studies: a systematic review and meta-analysis
Source: Eur J Nucl Med Mol Imaging. 2017 Aug 4;44(12):2105–16. doi: 10.1007/s00259-017-3775-4 (PMC5656693; doi:10.1007/s00259-017-3775-4)
Supplement: Supplementary file 2 — (DOCX 11 kb) [file 259_2017_3775_MOESM2_ESM.docx]

**Supplemental Table 2) Search strategy in Embase.com May 9 , 2016 (read from bottom-up).**

| **Set** | **Search terms** | **Result** |
| --- | --- | --- |
| #5 | #4 NOT 'conference abstract'/it | 335 |
| #4 | #1 AND #2 AND #3 | 614 |
| #3 | 'neoplasm'/exp OR 'oncology'/exp OR oncolog*:ab,ti OR cancer*:ab,ti OR neoplasm*:ab,ti OR tumour*:ab,ti OR tumor*:ab,ti OR carcinoma*:ab,ti OR malignan*:ab,ti OR metasta*:ab,ti OR lesion*:ab,ti OR lymphoma*:ab,ti | 5026012 |
| #2 | (partial NEXT/1 volume NEXT/1 effect*):ab,ti OR (partial NEXT/1 volume NEXT/1 correction*):ab,ti OR deconvolution*:ab,ti OR (recovery NEXT/1 coefficient*):ab,ti OR (point NEXT/1 spread NEXT/1 function NEXT/1 reconstruction*):ab,ti OR (psf NEXT/1 reconstruction*):ab,ti OR (resolution NEXT/1 model*):ab,ti OR (resolution NEXT/1 recover*):ab,ti OR (high NEXT/1 definition*):ab,ti OR (psf NEXT/1 model*):ab,ti OR (point NEXT/1 spread NEXT/1 function NEXT/1 model*):ab,ti OR (hd NEXT/1 reconstruction*):ab,ti | 13014 |
| #1 | 'positron emission tomography'/exp OR (positron NEXT/1 emission NEXT/1 tomograph*):ab,ti OR pet:ab,ti OR (pet NEXT/1 ct):ab,ti OR (fdg NEXT/1 pet*):ab,ti | 157749 |
