# Supplementary material for: Impact of partial-volume correction in oncological PET studies: a systematic review and meta-analysis
Source: Eur J Nucl Med Mol Imaging. 2017 Aug 4;44(12):2105–16. doi: 10.1007/s00259-017-3775-4 (PMC5656693; doi:10.1007/s00259-017-3775-4)
Supplement: Supplementary file 3 — (DOCX 72 kb) [file 259_2017_3775_MOESM3_ESM.docx]

**Supplemental Table 3) Technical PET details from included studies.** Reconstruction settings and resolution data pertain to the uncorrected images.

| **Ref.** | **SUV type** | **Delineation method** | **PVC method** | **Scanner** | **Reconstruction** | **system FWHM** | **filter FWHM** | **final FWHM** | **voxel-size** |
| --- | --- | --- | --- | --- | --- | --- | --- | --- | --- |
| [21] | mean | manual | RC | PET | FBP | - | - | - | - |
| [23] | mean | A50%max | RC | PET | MLA | - | - | - | 7*7*7 |
| [19] | max | manual | RC | PET | FBP | 6.5 | 10 (H) | - | - |
| [15] | max  mean (pvc) | manual | CT-volume correction | PET | RAMLA | - | - | - | 4*?*? |
| [16] | max | 90%max | RC | PET | OSEM (16s3i) | - | - | - | - |
| [17] | max | manual | RC | PET | OSEM (8s4i) | - | - | - | - |
| [18] | max  mean | manual | RC | PET/CT | OSEM (8s2i) | - | - | - | - |
| [22] | max | manual | RC | PET/CT | OSEM (12s4i) | 4.5 | 8 (G) | 9.2 | 2*?*? |
| [20] | max | manual | RC | PET | OSEM (28s2i) | 4.5 | 8 (G) | - | - |
| [24] | max | manual | RC | PET/CT | OSEM (14s2i) | - | - | - | - |
| [25] | max | manual | RC | PET | FBP | - | 12 (H) | - | - |
| [28] | max | manual | RC | PET | OSEM (28s2i) | - | 6 | - | 5.5*5.5*3.3 |
| [26] | max | manual | GTM | PET | OSEM | - | - | - | - |
| [31] | n.a. | n.a. | PSF reconstruction* | PET/CT | OSEM (8s4i) | - | 5 (G) | - | 4.1*4.1*4.1 |
| [34] | n.a. | n.a. | PSF reconstruction* | PET/CT | OSEM FORE | - | - | 4.2 | 2*?-? |
| [32] | n.a. | n.a. | PSF reconstruction* | PET/CT | OSEM (8s4i) | - | 5 (G) | 6.2 to 6.5 | 4.1*4.1*5 |
| [27] | max  mean | manual | IDC with denoising | PET/CT | RAMLA  OSEM (21s2i) | - | - | - | 4*4*4  4.7*4.7*3.3 |
| [29] | max | manual | RC | PET/CT | OSEM (15s2i) | - | 7 | - | 5.5*5.5*3.3 |
| [30] | mean | system-specific contrast-oriented algorithm | RC | PET/CT | OSEM (28s2i)  OSEM-PSF (21s3i) | - | 5.5  2 | - | - |
| [33] | n.a. | n.a. | PSF reconstruction* | PET/CT | OSEM (16s2i) | - | 6 (G) | - | 4.7*4.7*4.7 |
| [35] | max | scanner-implemented | RC | PET/CT | - | - | - | - | - |
| [37] | mean | A41%max | Mask-based PVC | PET/CT | OSEM (16s2i) | - | 5 (G) | - | - |
| [38] | max  mean  peak | fuzzy locally adaptive Bayesian  algorithm | IDC with denoising | PET/CT | RAMLA | - | 5 (G) | - | 4*4*4 |
| [36] | max | - | RC | PET | OSEM FORE (32s1i) | - | - | - | - |
| [39] | - | 60%max | RC | PET/CT | OSEM | - | - | - | 4.7*4.7*3.3 |
| [40] | mean | 40%max | RC | PET/CT | OSEM (28s2i) | - | - | - | - |
| [41] | n.a. | manual | RC | PET | - | - | - | 10 to 12 | - |
| [42] | max  mean  peak | fuzzy locally adaptive Bayesian  algorithm | IDC with denoising | PET/CT | RAMLA | - | - | - | 4*4*4 |
| [43] | max  mean | fuzzy locally adaptive Bayesian  algorithm | IDC with denoising | PET/CT | OSEM FORE (8s4i) | 6 | - | - | 4.1*4.1*3 |
| [44] | max  mean (pvc)  peak | system-specific contrast-oriented algorithm | RC + IDC | PET/CT | OSEM (28s2i) | - | 5.5 (G) | - | 3.9*3.9*4.3 |
| [45] | mean | adaptive iterative thresholding algorithm | RC | PET/CT | OSEM (8s4i) | - | 5 (G) | - | 4*4*2 |

RC = recovery coefficient; PSF = point spread function; CT = computed tomography; IDC = iterative deconvolution; GTM = geometric transfer matrix; FBP = filtered backprojection; MLA = maximum-likelihood algorithm; RAMLA = row-action maximum-likelihood algorithm; OSEM = ordered subset expectation maximization; FORE = Fourier rebinning; H = Hanning filter; G = Gauss filter; FWHM = full width at half maximum; n.a. = not applicable.
